# CD44-Deficiency Attenuates the Immunologic Responses to LPS and Delays the Onset of Endotoxic Shock-Induced Renal Inflammation and Dysfunction

**DOI:** 10.1371/journal.pone.0084479

**Published:** 2013-12-23

**Authors:** Elena Rampanelli, Mark C. Dessing, Nike Claessen, Gwendoline J. D. Teske, Sander P. J. Joosten, Steven T. Pals, Jaklien C. Leemans, Sandrine Florquin

**Affiliations:** 1 Department of Pathology, Academic Medical Center, University of Amsterdam, Amsterdam, The Netherlands; 2 Department of Pathology, Radboud University Nijmegen Center, Nijmegen, The Netherlands; University of Kentucky, United States of America

## Abstract

Acute kidney injury (AKI) is a common complication during systemic inflammatory response syndrome (SIRS), a potentially deadly clinical condition characterized by whole-body inflammatory state and organ dysfunction. CD44 is a ubiquitously expressed cell-surface transmembrane receptor with multiple functions in inflammatory processes, including sterile renal inflammation. The present study aimed to assess the role of CD44 in endotoxic shock-induced kidney inflammation and dysfunction by using CD44 KO and WT mice exposed intraperitoneally to LPS for 2, 4, and 24 hours . Upon LPS administration, CD44 expression in WT kidneys was augmented at all time-points. At 2 and 4 hours, CD44 KO animals showed a preserved renal function in comparison to WT mice. In absence of CD44, the pro-inflammatory cytokine levels in plasma and kidneys were lower, while renal expression of the anti-inflammatory cytokine IL-10 was higher. The cytokine levels were associated with decreased leukocyte influx and endothelial activation in CD44 KO kidneys. Furthermore, *in vitro* assays demonstrated a role of CD44 in enhancing macrophage cytokine responses to LPS and leukocyte migration. In conclusion, our study demonstrates that lack of CD44 impairs the early pro-inflammatory cytokine response to LPS, diminishes leukocyte migration/chemotaxis and endothelial activation, hence, delays endotoxic shock-induced AKI.

## Introduction

Systemic inflammatory response syndrome (SIRS) is a potentially deadly clinical condition associated with systemic activation of multiple inflammatory pathways that often results in severe organ dysfunction and failure, including acute renal failure (ARF) [1,2]. SIRS is frequently triggered by a primary localized infection (sepsis). Approximately 20-50% of septic patients diagnosed by positive blood culture develop ARF, and the combination of ARF and sepsis rises the mortality rate of septic patients from 30% to 70% [3].

Gram-negative bacteria account for about 60% of septic cases with a microbiological diagnosis, and the lipopolysaccharide (LPS) released from their outer membrane has a dominant role in initiating the inflammatory response [2]. Indeed, LPS has been largely used to induced SIRS condition in animal models and it is known to causes SIRS with ARF [4-6]. The principal mechanism by which LPS is sensed is *via* binding to LPS-binding protein (LBP) and CD14 and subsequently *via* signaling through Toll-like receptor-4 (TLR-4)-MD-2 complex [2]. LPS-induced TLR-4 activation promotes an early rise in pro-inflammatory cytokine production in many cell types, especially in mononuclear cells. The resulting cytokines such as tumor necrosis factor (TNF-α), interleukin (IL)-1 and IL-6, mediate renal injury directly or through the action of reactive oxygen/nitrogen species, caspases and nitric oxide (NO) [2,6] that causes renal vasoconstriction with sodium and water retention, which is the predominant pathogenic factor in early sepsis-related ARF [3].

Besides TLR-4, there are several other molecules involved in LPS-induced cell activation and the resulting SIRS. The adhesion molecule CD44 is a broadly distributed type I transmembrane glycoprotein receptor for hyaluronan (HA) that is constitutively expressed by hematopoietic and parenchymal cells [7]. A considerable number of studies showed a crucial role for CD44 in inflammatory disorders [8], including sterile renal inflammatory diseases [9-11]. Functions of CD44 in immune responses include leukocyte activation, adhesion and recruitment [8], direct interaction between bacteria and host cells [7,12], association with TLR-4 and MD-2 in HA recognition [13], activation of CD11b/CD18 receptor [14] and macrophage inhibitory factor (MIF) receptor CD74 signaling [15].

The role of CD44 in inflammation is complex and involves multiple cell types, ligands and signaling pathways. Therefore its involvement in bacterial host defense can vary depending on pathogen species/derivatives and primary infection site. This may explain why the literature on the role of CD44 in host defense is partially contradictory. For instance, in the absence of CD44 fewer macrophages migrate into lungs in response to inhaled LPS [16] and less macrophages and T lymphocytes are recruited into *Mycobacterium tuberculosis*-infected lungs in murine models [12]. In another murine model of polymicrobial sepsis, CD44 was shown to mediate pulmonary recruitment of neutrophils and the use of antibodies targeting CD44 inhibited lung damage [17]. In contrast, CD44-deficient mice display more lung inflammation and more pro-inflammatory cytokine release in *Escherichia coli*-induced pneumonia and peritonitis, respectively [18,19]. Yet, in an *E. coli*-induced urinary tract infection murine model, CD44-deficiency limits bacterial outgrowth without affecting neutrophils recruitment or cytokine production [20].

Despite extensive basic research and clinical studies, the pathophysiology of SIRS/sepsis is still poorly understood. Identification of new therapeutic targets for the management of septic shock remains imperative considering the high mortality rate in the face of standard treatment and of several clinical trials, including anti-TNF, anti-IL-1 and anti-TLR-4 therapies [2,21].

The present study aimed to assess the role of CD44 in the renal response to LPS-induced shock and to determine its function in LPS-induced activation of mononuclear cells.

## Materials and Methods

### *In vivo* experimental design

Eight to 12 weeks old pathogen-free male C57BL/6 wild-type (WT) mice and CD44-knockout (CD44 KO) mice on a C57BL/6 background [10,20] (n=8 per group) were injected intraperitoneally with 10µg/g body weight of LPS (*Escherichia coli* O111:B4, Sigma-Aldrich). Sham mice received saline solution. Two, 4 and 24 hours after LPS injection and 4 hours after saline solution injection, mice were sacrificed by cardiac exsanguination. Blood was drawn in heparinized tubes and half kidneys were snap-frozen in liquid nitrogen and half fixed in 10% formalin. 

### Ethics statement

The Institutional Animal Care and Use Committee of the University of Amsterdam approved all animal experiments.

### *In vitro* assays

Bone marrow-derived macrophages (BMM) were obtained by culturing freshly isolated BM cells in 10cm-diameter “bacteriological” plastic plates for 7 days in RPMI 1640 medium supplemented with 10% foetal calf serum (FCS) and 30% L929 cell conditioned medium, as source of murine macrophage colony-stimulating factor (M-CSF) [22]. BMM maturation was evaluated by FACS staining for F4/80 (Serotec): 97.3% of cells expressed this macrophage marker. BMM were cultured in RPMI 1640 medium with or without 100ng/ml LPS for 4 and 24 hours. CD44-cross ligation was induced by 30 minutes incubation with 5µg/ml IM7.8.1 antibodies (BD Pharmingen), followed by cross-linking with 5µg/ml rabbit IgG anti-rat (DAKO) for 20 minutes. Control cells were incubated with isotype control rat IgG2b and anti-rat IgG (both 5µg/ml, DAKO). The inhibitors SB203580 (10µM, Enzo Life Sciences), 3-Methyladenine (3-MA, 1mM, Enzo Life Sciences), Cli095 (0.5µg/ml, InvivoGen) were added to BMM 30 minutes prior to stimulation with LPS (100ng/ml) for 4 hours. The migration assay was performed with heparinized blood plated in a Transwell plate (5µm pore size membrane). Cells were loaded in 200µl 0% FCS-RPMI 1640 medium to the upper well; the lower chamber contained 700µl 0% FCS-RPMI 1640 medium with or without 200ng/ml macrophage inflammatory protein-2 (MIP-2) and 200ng/ml monocyte chemoattractant protein-1 (MCP-1). After 24 hours, cells were harvested from both upper and lower chamber and stained for CD11b and Ly6G (BD Pharmingen). 

### Immunohistochemistry, histological scoring and renal function

Renal tissues were fixed in 10% formalin for 24 hours and subsequently embedded in paraffin in a routine fashion. Immunohistochemistry stainings (immunostaining) were performed on 4µm renal sections using anti-CD44 (IM7.8.1, BD Pharmingen), anti-active caspase-3 (Cell Signaling), anti-Ly6G (BD Pharmingen), anti-F4/80 (Serotec), anti-CD3 (BD Pharmingen), anti-inducible NO synthase (iNOS, Abcam), anti-vascular cell adhesion molecule-1 (VCAM-1, R&D), anti-intercellular adhesion molecule-1 (ICAM-1, R&D), anti-phospho-AKT (Cell Signaling), anti-phospho-p38 MAPK (GeneTex). Hyaluronan (HA) was detected by biotinylated HA-binding protein (Calbiochem). Slides were developed using HRP-labeled secondary antibody (DAKO) and DAB (Sigma-Aldrich). Quantification of immunohistochemistry stainings were assessed in the cortex and cortico-medullary area: positive cells were counted per high power field (HPF, x20 magnification) or ten to fifteen high-power field (x20) pictures were taken per slide, and the positive areas were measured using ImageJ software (National Institute of Health, US) [23]. Results are shown as positive area in percentage of the total area analyzed. For assessing renal function, plasma urea concentration was measured by standard diagnostic procedure suitable for detection of samples of murine origin.

### ELISA

Heparinized blood was centrifuged at 10000 rpm for 10 minutes and plasma was harvested. Frozen kidneys were homogenized in lysis buffer (150mM NaCl, 15mM Tris, 1mM MgCl2 pH 7.4, 1mM CaCl_2_, 1% Triton) with addition of 1% protease inhibitor cocktail (P8340, Sigma). Specific ELISAs (R&D Systems) were utilized to measure IL-1β, IL-6, IL-10, MCP-1, TNF-α in plasma, kidney homogenates or cell supernatant.

### Quantitative real-time PCR

Total RNA was extracted from 10 frozen renal sections (30µm thick) or from *in vitro* cultured cells with Trizol reagent (Invitrogen). RNA was converted to cDNA using oligo-dT primers. Quantitative real-time PCR (Q–PCR) was performed on a LightCycler® 480 System (Roche) using SYBR Green-SensiMix (Bioline). SYBR green dye intensity was analyzed with linear regression analysis. Transcript expression was normalized towards the housekeeping gene TATA-box binding protein (TBP). Amplified genes and primers sequences are as follows: TBP forward 5’-caggagccaagagtgaagaac reverse 5’-ggaaataattctggctcatagctact, CD44-pan forward 5’-tccgaattagctggacactc reverse 5’-ccacaccttctcctactattgac, TLR-4 forward 5’-ggactctgatcatggcactg reverse 5’-ctgatccatgcattggtaggt, kidney injury molecule-1 (KIM-1) forward 5’-tggttgccttccgtgtctct reverse 5’-tcagctcgggaatgcacaa, neutrophil gelatinase-associated lipocalin (NGAL) forward 5’-gcctcaaggacgacaacatc reverse 5’-ctgaaccattgggtctctgc, VCAM-1 forward 5’-gacgattccggcatttatgt reverse 5’-ttttggagagacttggataatca, ICAM-1 forward 5’-cttctgagcggcgtcgagcc reverse 5’-gccgaggaccatacagcacgt, IL-1β forward 5’-tgagcaccttcttttccttca reverse 5’-ttgtctaatgggaacgtcacac, interferon-β (IFNβ) forward 5’-ggaaagattgacgtgggaga reverse 5’-cctttgcaccctccagtaat, CD11b forward 5’-aaggatgctggggaggtc reverse 5’-gtcataagtgacagtgctctgga. All primers were manufactured by Biolegio.

### Immunoblotting

Cells were incubated at 4°C for 30 minutes in RIPA buffer containing 20mM Tris-HCl pH7.5, 150mM NaCl, 5mM EDTA, 1% NaDOC, 1% NP-40, 200µM Na_3_VO_4_, 50mM NaF, 1% protease inhibitor cocktail (P8340, Sigma), 0.1% SDS, 10% glycerol. After centrifuging at 14000 g, the supernatants were collected and nuclei were incubated with RIPA buffer containing 2% SDS and 20% glycerol and afterwards sonicated for 20 seconds. Lysates were subjected to western-blot (WB) analysis using anti-phospho-IκBα, anti-NF-κB p65, anti-MD-2 (Enzo Life Sciences), anti-MyD88 (Cell Signaling), anti-Trif (Abcam), anti-TRAF6 (Santa Cruz Biotechnology). HRP-conjugated secondary antibodies (DAKO) were used and HRP activity was visualized with ECL-reagent (GE Healthcare). β-actin (Abcam) and lamin A/C (Cell Signaling) were used as loading controls for cytoplasmic and nuclear fractions, respectively. Densitometric quantification analysis was performed on imagines of scanned films using the ImageJ software.

### Flow cytometry

Following erythrocytes disruption with lysis buffer (160mM NH4Cl, 10mM KHCO3 and 0.1mM EDTA, pH 7.4), blood cells were stained with monoclonal anti-Ly6-FITC-conjugated (BD Pharmingen), anti-CD11b-APC-conjugated (BD Pharmingen) or anti-F4/80-FITC-conjugated (Serotec). Before analysis, cells were fixed in PBS containing 2% paraformaldehyde. Staining was visualized on a FACS Canto II (BD Biosciences) and analysis was done using FlowJo software (Tree Star).

### Data analysis

Statistical analysis was performed using Mann-Whitney U test or Student t test. Data are shown as mean and standard error of the mean (SEM); *P* < 0.05 was considered to be significant. 

## Results

### Lower levels of MCP-1 and IL-1β in plasma of CD44 KO mice after LPS injection

**Figure 1:**
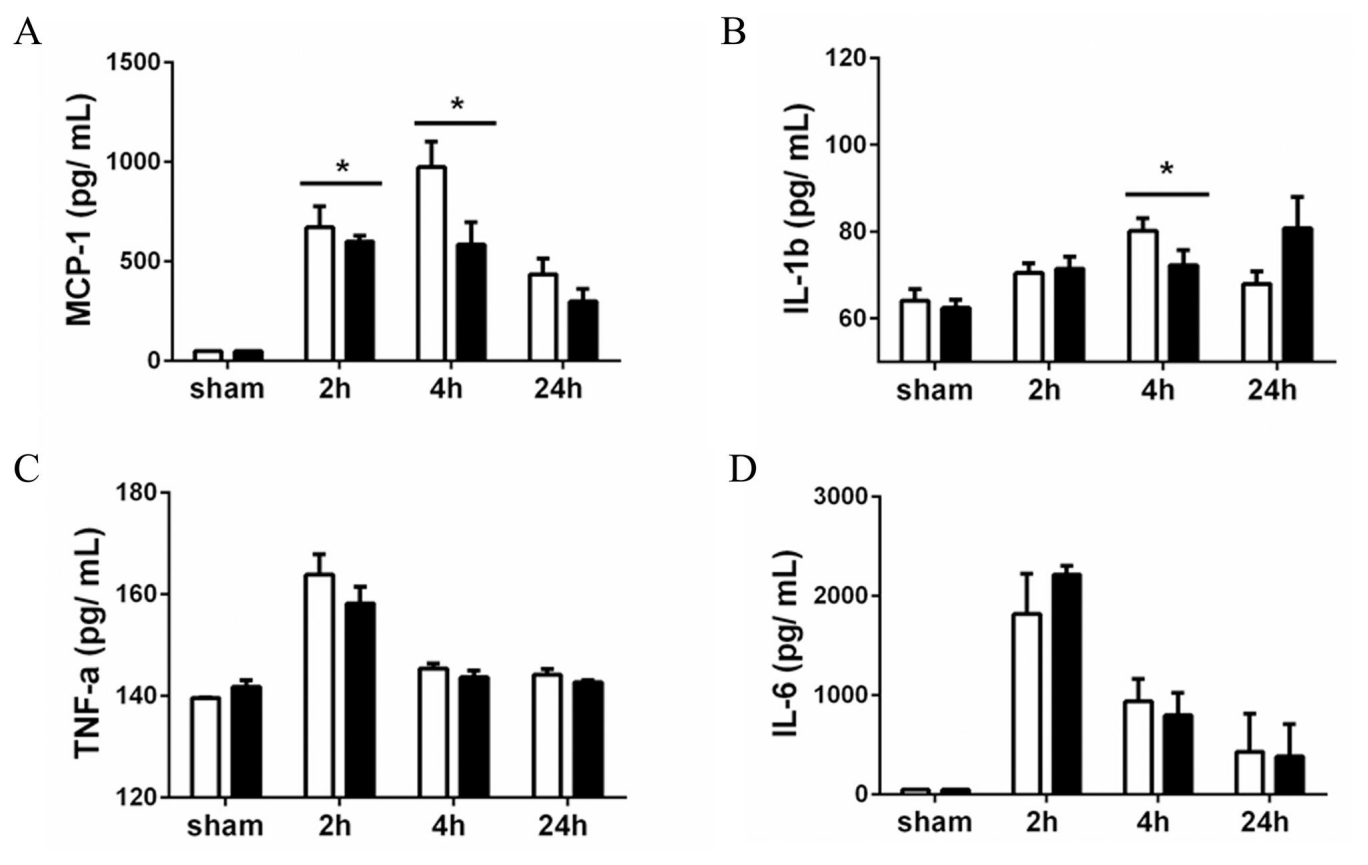
Systemic inflammation. Concentration (pg/ml) of (**A**) MCP-1, (**B**) IL-1β, (**C**) TNF-α, (**D**) IL-6 in plasma of WT (white bars) and CD44 KO (black bars) mice assessed by ELISA. Data expressed as mean + SEM, n=8, *=p<0.05.

The hallmark of endotoxin-shock is a systemic inflammatory response. We therefore measured the blood levels of several pro-inflammatory cytokines. Within 2 hours after LPS injection, all analyzed cytokines were elevated in blood (Figure 1). Compared to WT mice however, CD44 KO animals showed less MCP-1 (2 and 4 hours) and less IL-1β (4 hours), whereas TNF-α and IL-6 serum levels were equal between WT and CD44 KO mice. Of note, blood cells from both strains express equal mRNA levels of TLR-4 in physiological condition (data not shown).

### Increased renal expression of CD44 and its ligand hyaluronan upon LPS injection

**Figure 2:**
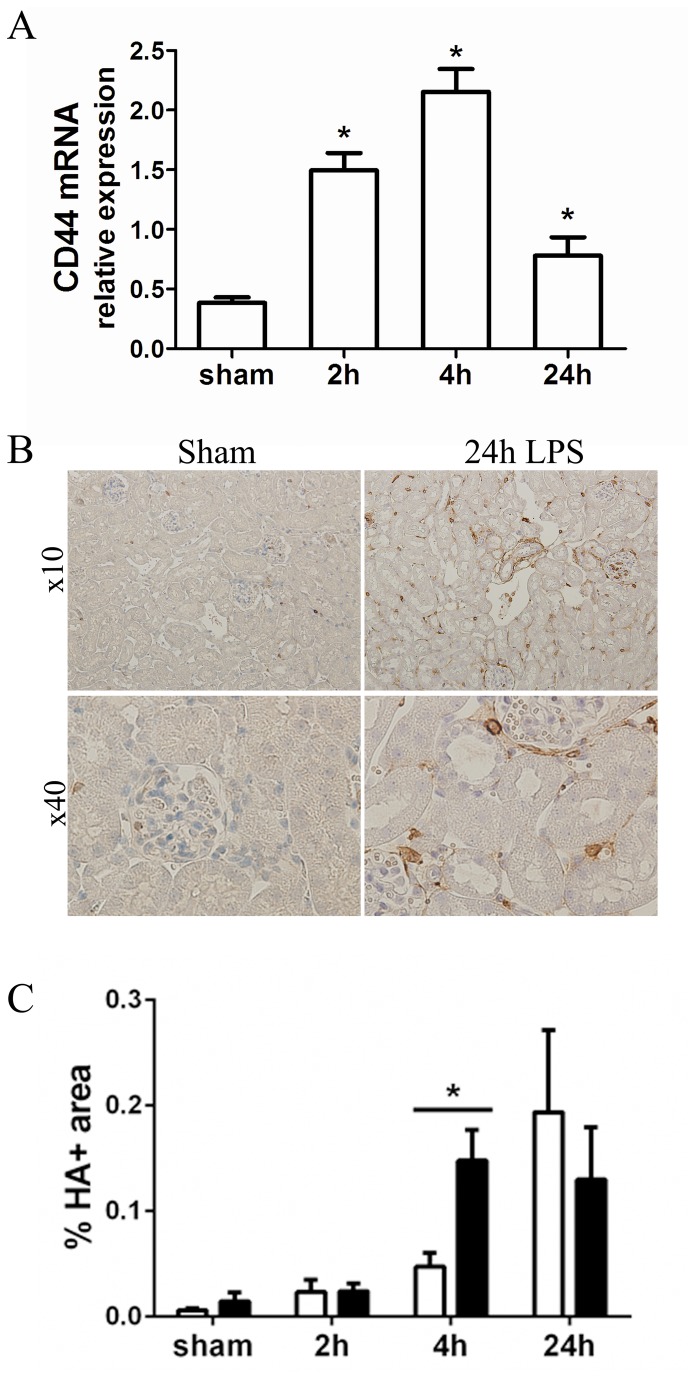
Renal CD44 and HA expression. (**A**) Expression of CD44 in kidneys in saline- and LPS-treated WT mice assessed by Q-PCR. Data normalized for TBP transcript expression. Mean + SEM, n=8, *=p<0.05 versus (vs) sham. (**B**) Representative micrographs (x10 magnification upper panel, x40 lower panel) of CD44 immunostaining in renal sections of WT sham and 24 hours LPS-treated mice. (**C**) Quantification of HABP immunostaining in kidneys of WT (white bars) and CD44 KO (black bars) mice. Data expressed as percent positive area of the total area analyzed. Mean + SEM, n=8, *=p<0.05.

We next assessed the expression of CD44 by Q-PCR and immunostaining in WT and CD44 KO kidneys. Q-PCR analysis showed an increase at 2, 4 and 24 hours in CD44 transcripts in WT kidneys (Figure 2A). Immunohistochemistry staining confirmed an increase in CD44-positive cells in WT kidneys after LPS administration (Figure 2B). CD44 expressing cells were found mainly in the interstitium and some in the glomeruli. In contrast to what is seen in other renal pathological conditions [9,24,25], CD44 was absent on tubular epithelial cells (TEC) at all time-points.

Hyaluronan (HA) is a major component of the extracellular matrix (ECM) and a major ligand of CD44. Fragments of HA are generated during inflammation or injury, and they can serve as endogenous triggers of TLR signaling and promote cytokine production [26]. LPS induced an accumulation of renal interstitial HA, which picked at 24 hours in WT kidneys and at 4 hours in CD44 KO kidneys (Figure 2C).

Together with CD44 and HA, we measured the renal expression of the LPS/HA receptor TLR-4 [2,26], which showed no differences between the strains in control situation and was enhanced as early as 2 hours post-injection in WT kidneys and later, at 4 hours, in CD44 KO kidneys (data not shown).

### Preserved renal function in CD44 KO mice

**Figure 3:**
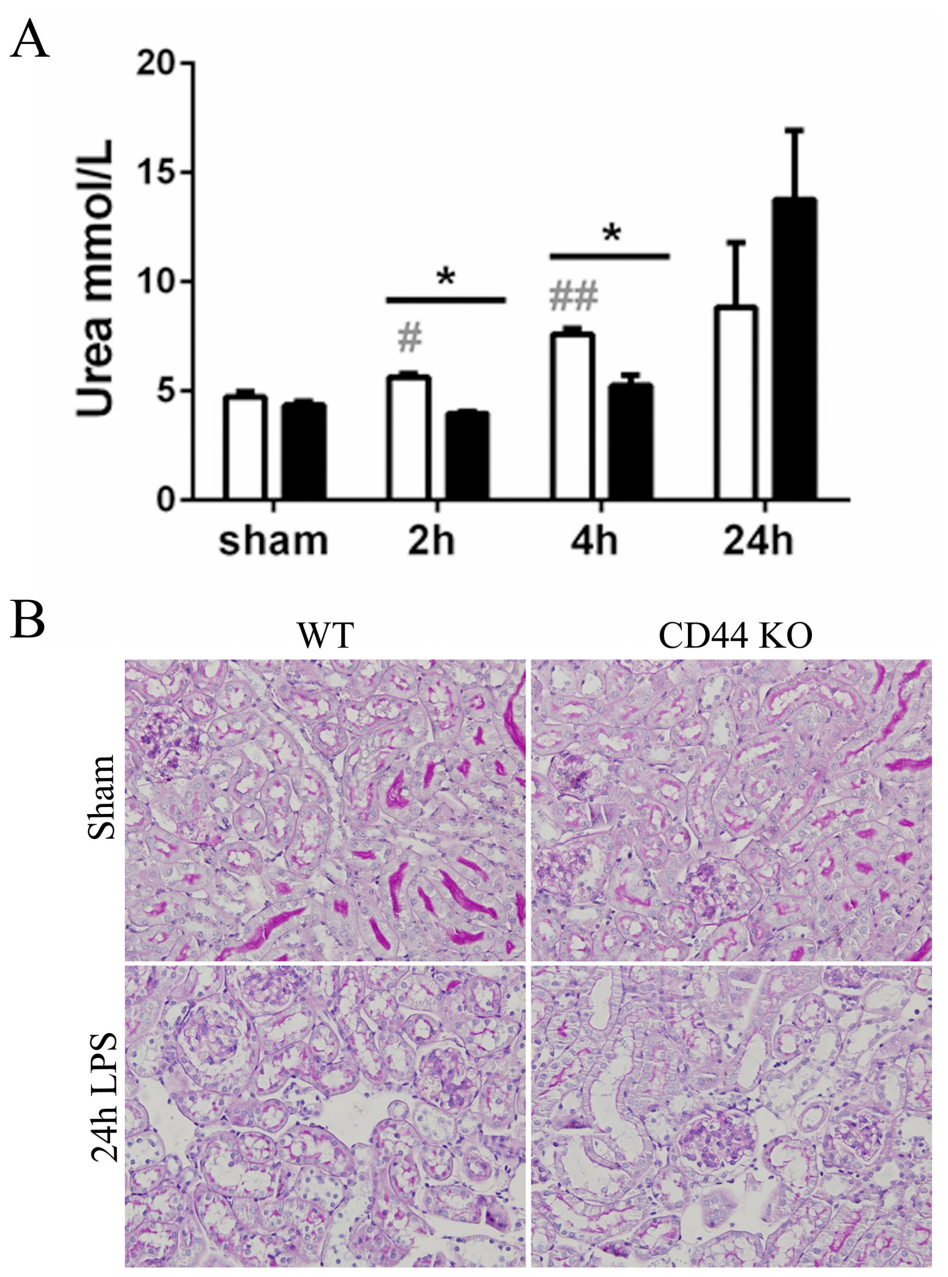
Renal injury. (**A**) Renal function assessed by measurement of urea levels (mmol/L) in plasma of WT (white bars) and CD44 KO (black bars) mice. Mean + SEM, n=8, *=p<0.05, WT vs CD44 KO; #=p<0.05, ##=P<0.01, vs sham. (**B**) Representative micrographs (x20) of Periodic acid-Schiff Diastase (PAS-D) stained renal sections of saline- and 24 hours LPS-treated WT and CD44 KO mice.

Renal function was assessed 2, 4 and 24 hours after LPS administration by measuring plasma urea levels (Figure 3A). Blood urea levels picked at 24 hours in both mice strains, but WT mice showed a compromised renal function as early as 2 hours after LPS injection, whereas CD44 KO mice maintained renal function at 2 and 4 hours as compared to saline-treated animals.

LPS caused relatively mild morphological changes at 24 hours in renal tissues, including loss of brush border, tubular vacuolization and dilatation, but no necrosis or cast formation [6] (Figure 3B). These morphological alteration were similar in both mice groups, and so was the gene expression of the early markers of tubular injury KIM-1 and NGAL [27], which gradually increased in time after LPS administration in a similar manner in WT and CD44 KO kidneys. (Figure S1A,B). Upon LPS challenge, the apoptosis rate of tubular cells was low and comparable between the mice strains (Figure S1C).

### Less renal cytokine release in LPS-treated CD44 KO mice

**Figure 4:**
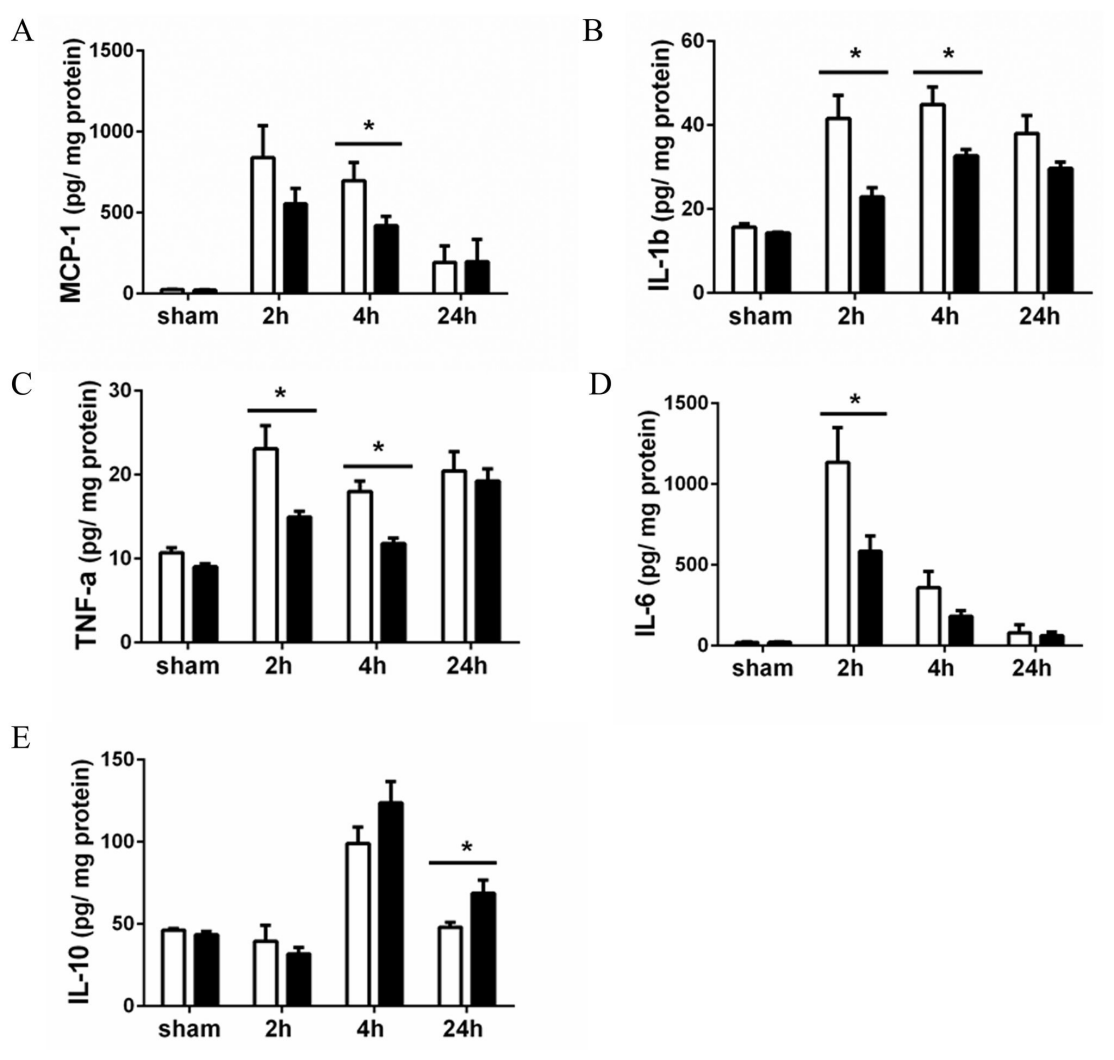
Cytokines in the kidneys. Expression (pg/mg proteins) of (**A**) MCP-1, (**B**) IL-1β, (**C**) TNF-α, (**D**) IL-6 and (**E**) IL-10 in kidneys of WT (white bars) and CD44 KO (black bars) mice measured by ELISA. Data shown as mean + SEM, n=8, *=p<0.05.

Next, we assessed the inflammatory state in the kidneys by measuring local cytokine levels. LPS administration led to a rapid increase (2 hours) in renal production of pro-inflammatory cytokines (MCP-1, IL-1β, TNF-α, IL-6), which were generally higher at 2 and 4 hours in WT kidneys as compared to CD44 KO kidneys (Figure 4A-D). As a mechanism to counteract excessive inflammation, anti-inflammatory cytokines are produced after endotoxin stimulation [21]. Indeed, the renal levels of the anti-inflammatory IL-10 greatly increased at 4h after LPS injection in both strains and were significantly higher in CD44 KO kidneys at 24 hours in respect to WT kidneys (Figure 4E). 

### Reduced leukocyte influx into CD44 KO kidneys upon LPS

**Figure 5:**
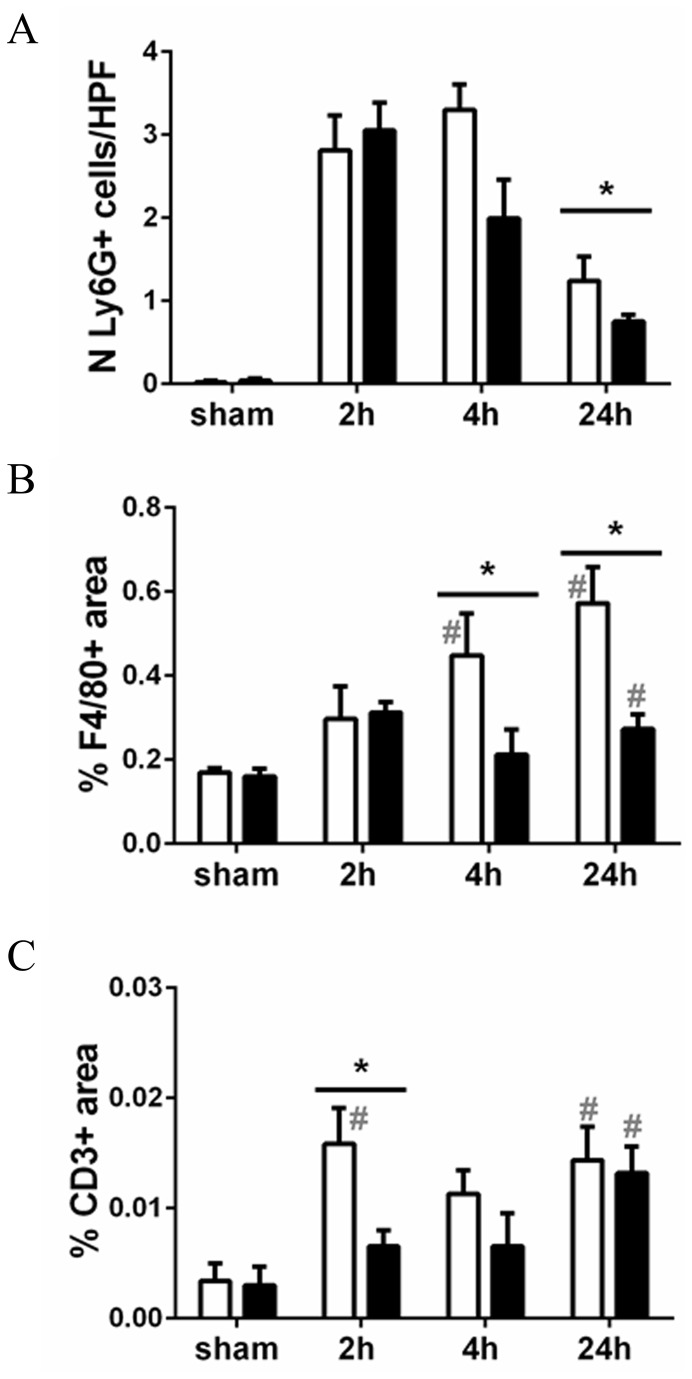
Leukocyte influx in the kidneys. Quantifications of immunostainings for detection of (**A**) granulocytes, (**B**) macrophages, (**C**) T lymphocytes in paraffin renal sections of WT (white bars) and CD44 KO (black bars) mice. (**A**) Number of Ly6G-positive cells per HPF (x20). (**B**,**C**) Data expressed as percent positive area of the total areal analyzed. Mean + SEM, n=8, *=p<0.05, WT vs CD44 KO; #=p<0.05, vs sham; in (**A**) all groups # vs sham.

Considering the differences in cytokine expression between WT and CD44 KO mice, we wondered whether the recruitment of inflammatory cells would be affected in absence of CD44. The presence of granulocytes, macrophages and T lymphocytes in the kidneys was detected by immunohistochemistry (Figure 5A-C). The CD44 KO kidneys showed less granulocytes at 24 hours, less macrophages at 4 and 24 hours, and less T lymphocytes at 2 hours as compared to WT kidneys.

### Attenuated endothelial activation in CD44 KO kidneys

**Figure 6:**
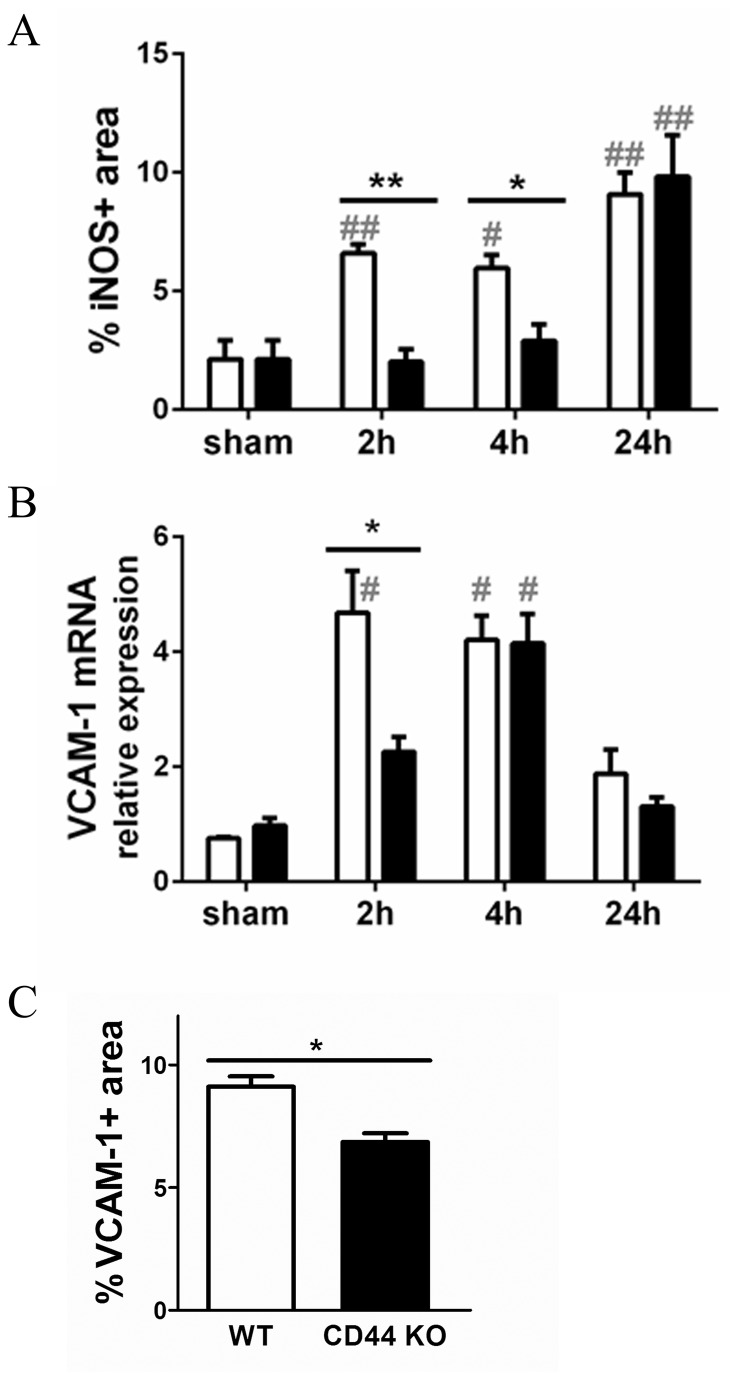
Renal endothelial activation. (**A**) iNOS expression detected by immunostaining in paraffin renal sections of WT (white bars) and CD44 KO (black bars); quantification shown as positive area percentage of total area. (**B**) Q-PCR analysis for renal expression of VCAM-1; data normalized for TBP expression levels. (**C**) Digital analysis of VCAM-1 protein expression 2 hours after LPS injection, assessed by immunohistochemistry on frozen renal sections. Results expressed as positive area percentage of total area. Mean + SEM, n=8, *=p<0.05 **=p<0.01, WT vs CD44 KO; #=p<0.05, ##=P<0.01, vs sham.

The dissimilarities in renal influx of inflammatory cells could be attributed to the disparity in cytokine levels, but also to a differential endothelial inflammatory/activation state. Endotoxic shock is known to enhance the levels of VCAM-1 and ICAM-1, which indicate endothelial activation, and to induce NO synthesis, which decreases the systemic vascular resistance [6]. In WT mice, LPS administration rapidly (at 2 hours) augmented the renal levels of iNOS protein (Figure 6A), VCAM-1 (Figure 6B) and ICAM-1 mRNAs (Figure S1D). In CD44 KO kidneys, expression of iNOS was significantly lower at 2 and 4 hours in respect to WT kidneys (Figure 6A). Furthermore, at 2 hours CD44 KO kidneys revealed less VCAM-1 transcript expression (Figure 6B) and a trend (p=0.053) toward less ICAM-1 transcript expression (Figure S1D). Immunohistochemistry confirmed lower levels of VCAM-1 protein in CD44 KO kidneys at 2 hours (Figure 6C); instead, no significant differences were found in ICAM-1 protein levels 2 hours after LPS injection (Figure S1E).

### Diminished cytokine release and TLR-4-signaling in LPS-stimulated CD44 KO macrophages

**Figure 7:**
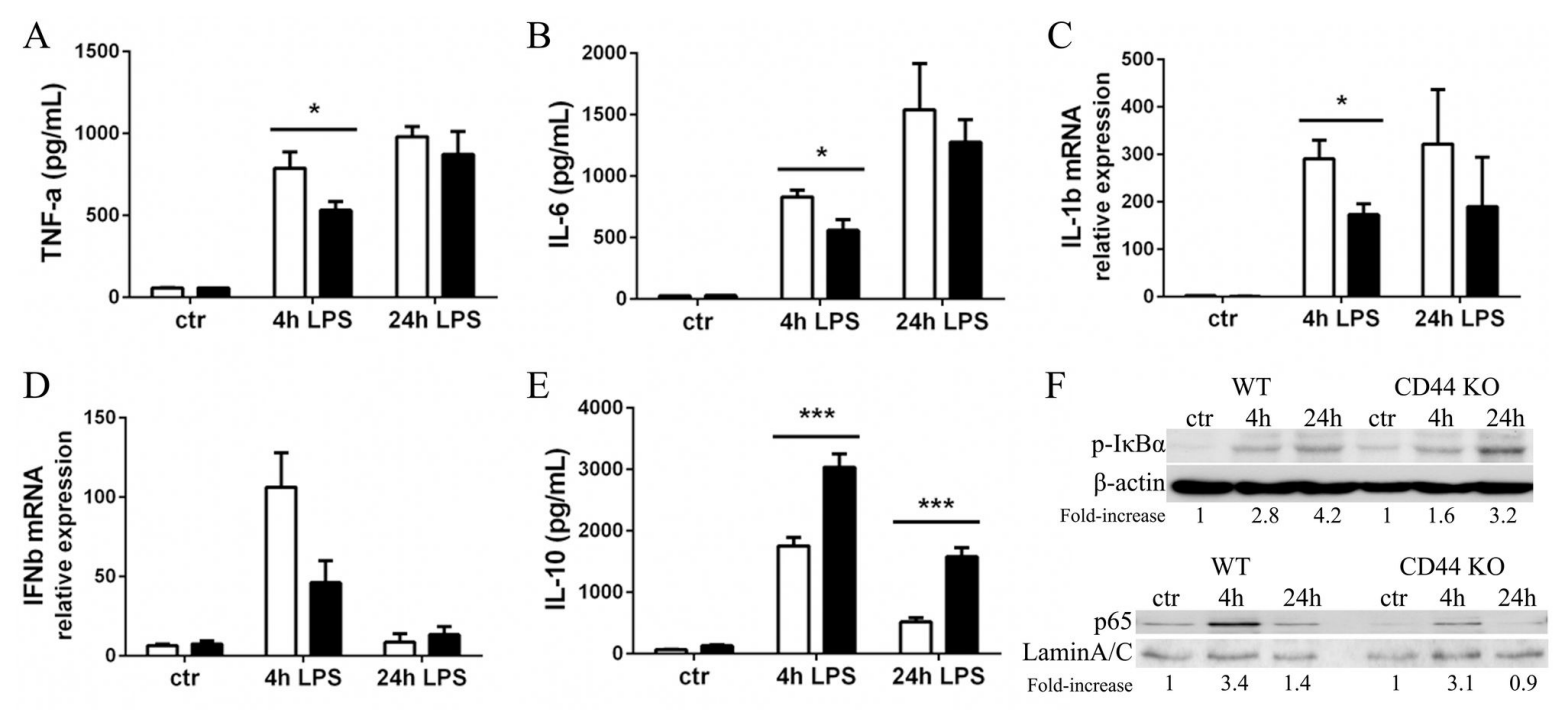
Macrophage response to LPS in presence/absence of CD44. *In* *vitro* LPS (100ng/ml) stimulation of WT (white bars) and CD44 KO (black bars) bone marrow-derived macrophages (BMM) for 4 and 24 hours. Secretion levels (pg/ml) of (**A**) TNF-α and (**B**) IL-6 detected by ELISA. (**C**) Gene expression of IL-1β and (**D**) IFNβ normalized for TBP transcript levels, assessed by Q-PCR. (**E**) ELISA measurement of IL-10 supernatant concentration (pg/ml). Mean + SEM, n=10, *=p<0.05, ***=p<0.001. (**F**) WB assay of cell lysates from WT and CD44 KO BMM for detection of cytoplasmatic phosphorylated (p)-IκBα and nuclear p65. β-actin or LaminA/C used as loading controls. Fold-increase in protein expression in respect to control is indicated.

To obtain better insight into the role of CD44 in the primary cell response to LPS, we stimulated bone marrow-derived macrophages from WT and CD44 KO mice with 100ng/ml LPS for 4 and 24 hours. In line with the *in vivo* results, CD44 KO macrophages secreted significantly less TNF-α and IL-6 after 4 hours stimulation, while after 24 hours the pro-inflammatory cytokine production was equal between the two groups (Figure 7A,B). In addition, the gene expression of IL-1β (p=0.014) and IFNβ (p=0.056) was lower in CD44 KO macrophages as compared to WT macrophages after 4 hours of stimulation (Figure 7C,D). On the contrary, the amount of secreted IL-10 was higher in the supernatant of CD44 KO cells at both 4 and 24 hours of stimulation (Figure 7E). TLR-4 activates the MyD88-dependent and Trif-dependent pathways. The first one leads to activation of NF-κB and AP-1 inducing several pro-inflammatory cytokines, while the second pathway leads to IRF3 activation and hence induction of type I IFN, particularly IFNβ [28]. The lower expression of pro-inflammatory cytokines, including IFNβ, by CD44 KO macrophages suggests that both MyD88- and Trif-dependent pathways are less activated in these cells. To determine if this phenotype was due to lower expression levels of the molecules involved in sensing LPS and in mediating the intracellular pathways, we assessed in WT and CD44 KO macrophages mRNA levels of the LPS sensors TLR-4 and CD11b, and protein levels of MD-2, MyD88, Trif and TRAF6, another adaptor protein of the TLR-pathway [28]. None of these molecules showed differential expression in WT and CD44 KO cells (data not shown). To prove the activation of NF-κB, the phosphorylation of the inhibitor of NF-κB (IκBα) and the nuclear translocation of the NF-κB p65-subunit were evaluated (Figure 7F). Upon LPS-stimulation both phosphorylated IκBα and p65 were increased, respectively, in the cytoplasmatic and nuclear fractions. Relatively to control, the fold-increase, at 4 and 24 hours, in phosphorylated IκBα was equal to 2.8 and 4.2 in WT BMM, and to 1.6 and 3.2 in CD44 KO cells. WT macrophages displayed a 3.4 (4 hours) and a 1.4 (24 hours) fold-increase in nuclear p65, whereas in CD44 KO BMM p65 was augmented only at 4 hours (3.1 fold-increase).

**Figure 8:**
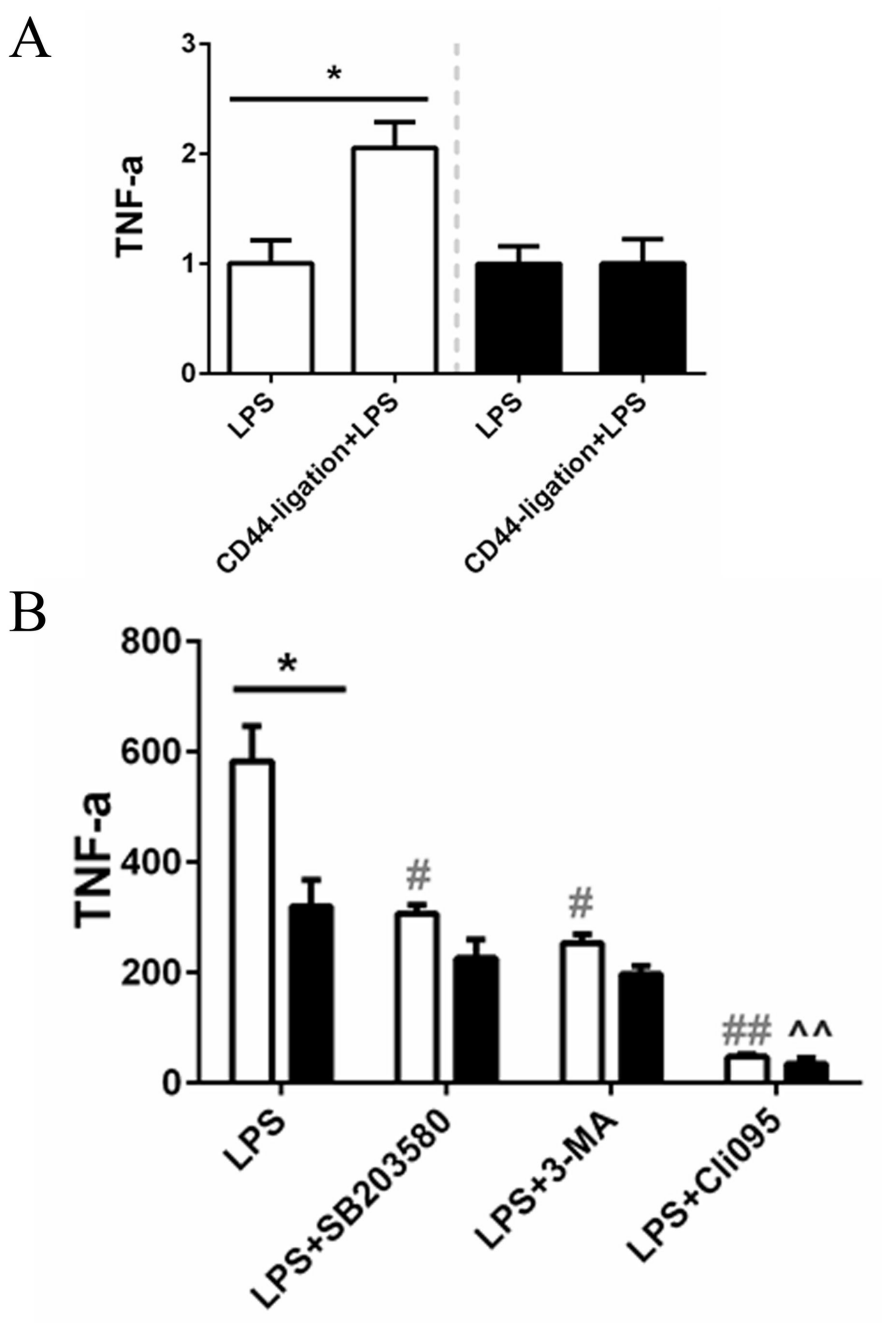
LPS-challenged macrophages after CD44-ligation and inhibitors pre-treatment. *In* *vitro* LPS (100ng/ml) stimulation of WT (white bars) and CD44 KO (black bars) BMM for 4 hours after CD44-ligation. (**A**) ELISA for detection of TNF-α in supernatants; data shown as fold-increase compared to LPS-stimulated BMM. (**B**) TNF-α levels (pg/ml) in presence or not of 10µM SB203580, 10mM 3-MA, or 0.5ug/ml Cli095 (ELISA). Mean + SEM, n=6, *=p<0.05, WT vs CD44 KO; #=p<0.05, ##=p<0.01, vs WT+LPS (no inhibitors); ^^=p<0.01, vs CD44 KO+LPS (no inhibitors).

We next wondered whether CD44-activation by ligation would affect the cell response to LPS in terms of cytokine production. In WT macrophages CD44 ligation induced a further increase in TNF-α secretion in response to LPS (Figure 8A); this effect was absent in CD44 KO cells. In WT BMM ligation of CD44 on itself did not increase cytokine secretion (data not shown). CD44 mediates activation of several pathways, including mitogen-activated protein kinases (MAPK) and phosphoinositide 3-OH kinase (PI3K)/Akt pathways [29], which have been shown to mediate LPS-elicited NF-κB activation [28,30-33]. Blocking p38 MAPK with SB203580 and PI3K with 3-MA, prior to LPS-exposure, significantly diminished TNF-α secretion in WT macrophages and not in CD44 KO BMM (Figure 8B). After treatment with the inhibitors, WT BMM cytokine-secretion was reduced to the levels of CD44 KO BMM stimulated with LPS alone. Inhibition of TLR4 with Cli095, instead, blocked completely cytokine-secretion in both cell groups. These findings suggest that CD44 is not completely necessary for TLR-4-activation, whereas is required for the LPS-induced p38 MAPK and PI3K signaling.

Finally, we evaluated renal expression of phosphorylated Akt and p38 MAPK by immunohistochemistry (Figure S1F,G). No statistically significant differences were found, presumably because of the high expression of both proteins in their phosphorylated state in tubular cells. Given that WT TEC lack CD44, they behave similarly to CD44 KO TEC, as previously shown by morphological observation, analysis of tubular injury markers and TEC apoptosis rate.

### Impaired migration of CD44 KO monocytes and granulocytes

**Figure 9:**
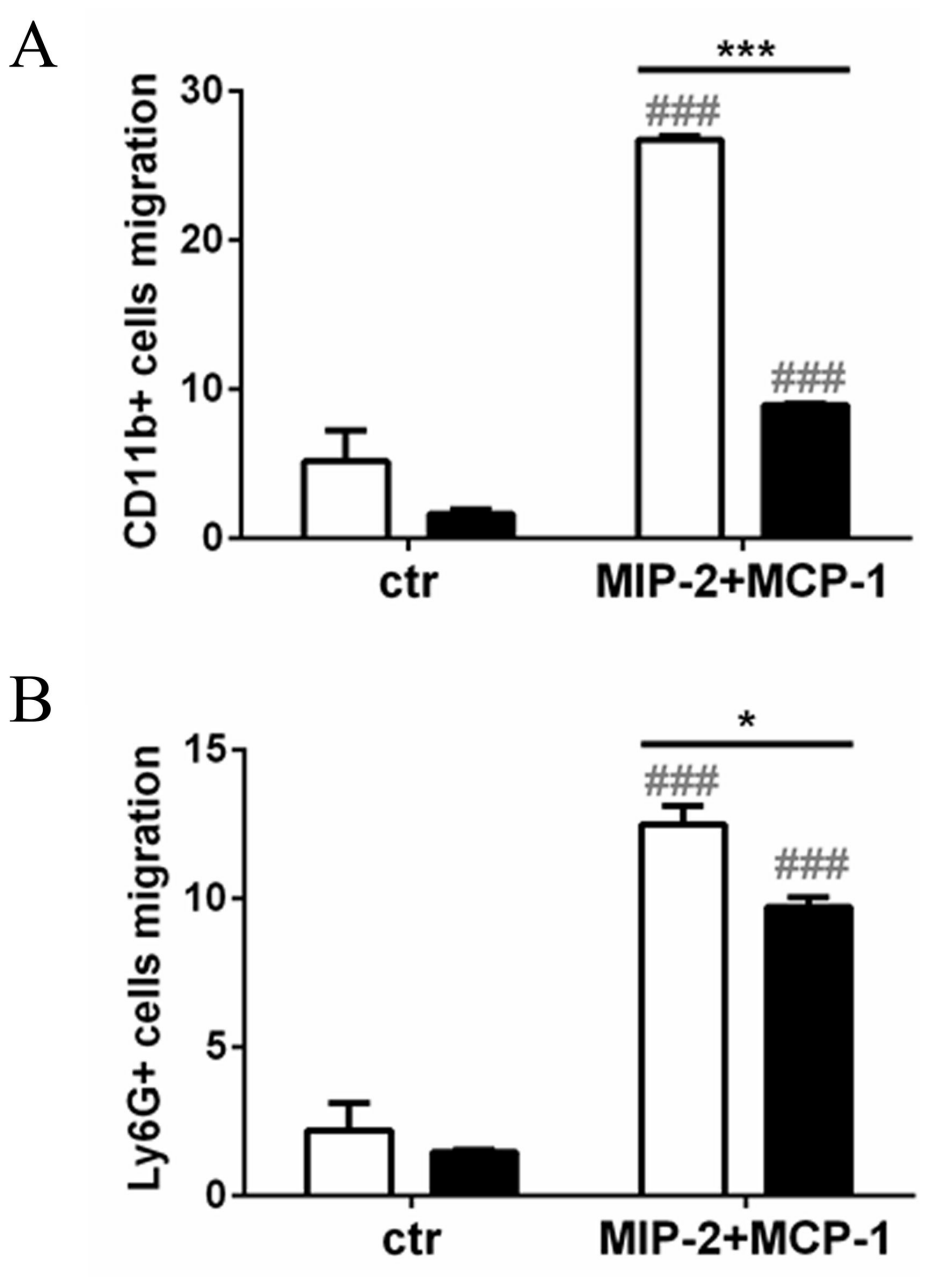
Migratory ability of blood leukocytes in absence of CD44. Transwell migration assay of (**A**) monocytes and (**B**) granulocytes from WT (white bars) and CD44 KO (black bars) blood. Immunofluorescent staining for flow cytometric analysis of (**A**) CD11b and (**B**) Ly6G after 24 hours culture in Transwell plate with or without 200ng/ml MIP-2 and MCP-1 in the lower chamber. Data expressed as percent cells in the lower chamber of the total number of cells. Mean + SEM, n=3, *=p<0.05, WT vs CD44 KO; ###=p<0.001, vs control.

Since less inflammatory cell influx was found in CD44 KO kidneys *in vivo* and CD44 is known to play a role in cell migration, the migratory ability of LPS-treated blood cells from WT and CD44 KO mice was investigated by Transwell migration assay. In absence of CD44, a lower number of CD11b- and Ly6G-positive cells migrated into the lower chamber containing medium supplemented with MIP-2 and MCP-1 after 24 hours exposure (Figure 9).

## Discussion

Sepsis is a major public health problem; it is the leading cause of death in non-coronary intensive care units and is the 11^th^ cause of death in the United States. Therefore, better understanding of the pathophysiology of sepsis and identification of new targets is very needed to improve septic shock treatment [34]. Our study demonstrates that upon LPS exposure, lack of CD44 impairs the early pro-inflammatory cytokine response, inflammatory cell migration/chemotaxis, endothelial activation, and therefore delays the onset of endotoxin shock-induced renal dysfunction.

Since sepsis is defined as a systemic inflammatory response syndrome, we evaluated the plasma pro-inflammatory cytokine levels, which are known to be upregulated in septic patients and correlate with mortality [2,35], and the cytokine expression in kidneys. In both compartments, and particularly in kidneys, the levels of pro-inflammatory cytokines were lower in CD44-deficient mice. The fact that cytokine expression in kidneys presents greater differences between the mice strains compared to plasma cytokine levels can be attributed to the secondary amplification of inflammation. Through extrarenal TLR-4, LPS causes an early rise in cytokines, which in turn activate a second level of inflammatory cascade including further cytokine production, NO synthesis, upregulation of cell adhesion molecules that results in inflammatory cells migration into tissues, endothelial injury and organ damage [2]. Hence, a relative small difference in the cytokine plasma levels may produce a greater effect in organs. This could result from the CD44 interaction with molecules present in circulation or ECM, such as HA. Hyaluronan, is a major CD44 ligand capable of inducing cytokine production [26]. After LPS administration, HA was upregulated in renal interstitium and expressed at higher levels in CD44 KO kidneys at 4 hours as compared to WT. This difference can be attributed to the role of CD44 in HA cellular uptake [26]. In circulation, CD44 may interact with cytokines such as macrophage inflammatory protein-1β (MIP-1β) [36] mediating lymphocyte-endothelial adhesion and/or with the MIF-CD74 receptor complex [15] to initiate signal transduction. Indeed, Shi et al. demonstrated that CD44 is required for signaling downstream of MIF, which is an important cytokine of septic shock as its neutralization or disruption of its activity improves survival in experimental sepsis [15,21]. The lower cytokine levels might partially explain the poorer recruitment of inflammatory cells into CD44 KO kidneys. CD44 is known to mediate macrophage- and lymphocyte-endothelial cell adhesion [8], thus it is reasonable to assume that WT leukocytes can more easily adhere to endothelium and extravasate. Our study shows a contribution of CD44 not only in leukocyte migration into renal parenchyma, but also in chemotaxis of monocytes and granulocytes *in vitro*. Besides its function in leukocyte adhesion and rolling, CD44 plays a role in cell migration also at the intracellular level. Indeed, CD44 cytoplasmic tail interacts with the actin cytoskeleton through ankyrin and erzin, radixin, moesin (ERM proteins), which are engaged in cell migration. After interacting with the cytoskeletal linker proteins, CD44 is guided to the leading edge of cells, promoting migration, and the activation-induced association with integrins has an essential role [29]. Another cause of impaired inflammatory cell influx in CD44 KO kidneys could be the lessened renal upregulation at 2 hours of VCAM-1, which mediates leukocyte-endothelial cell adhesion. Interestingly, we also found profound differences in renal iNOS, a mediator of capillary dysfunction and renal injury induced by LPS [6,37]. In the absence of CD44, renal iNOS expression remained at basal levels at 2 and 4 hours and was significantly increased only at 24 hours, whereas the WT kidneys displayed a statistically significant increase of iNOS 2 hours after LPS administration. Wu et al. showed that following LPS administration peritubular capillary dysfunction is an early event that induces tubular stress and precedes renal failure and the time course of capillary dysfunction is paralleled by the induction of iNOS in the kidney [6]. Indeed, the pattern of iNOS expression reflects the plasmatic urea rate, which did not increase until 24 hours in CD44 KO mice. Altogether, the lower inflammatory cytokine levels, the lower endothelial activation state, the lower iNOS induction, and hence the reduced inflammatory cell influx and renal inflammation state contribute to the delayed onset of renal dysfunction in CD44-deficient mice. The absence of CD44 on TEC before and during endotoxemia may explain the comparable levels of tubular injury markers KIM-1 and NGAL [27] and TEC apoptosis between the groups, and suggest that the phenotype observed is due to CD44 expression by inflammatory cells and endothelial cells and not by renal tubular cells.

To better understand the phenotype seen *in vivo* and to investigate whether CD44 serves a role mainly in the primary cell response to LPS or in the secondary amplification of the inflammatory cascade, we performed several *in vitro* assays using bone-marrow derived macrophages. Upon 4 hours LPS-stimulation CD44 KO macrophages secreted less IL-6 and TNF-α proteins and expressed less IL-1β and IFNβ mRNAs than WT BMM, suggesting that both the MyD88- and Trif-TLR-4-dowstream pathways are affected by CD44-deficiency. These results support the *in vivo* data and were associated with diminished activation of the NF-κB pathway in CD44 KO BMM. The *in vitro* results suggest that CD44 can directly modify the biologic response to LPS in macrophages.

Strikingly, both *in vivo* and *in vitro* more anti-inflammatory IL-10 is produced by CD44 KO cells, besides lower levels of pro-inflammatory cytokines. A possible/plausible regulator of this phenomenon is heme oxygenase-1 (HO-1), which is induced by LPS and provides defense against endotoxemia, including LPS-induced ARF, controlling the IL-6/IL-10 balance [38,39]. Indeed, renal expression of HO-1 was enhanced after LPS-injection, and at 24 hours HO-1 mRNAs and proteins were significantly higher in CD44 KO kidneys compared to WT (data not shown). 

Ligation of CD44 triggered a significantly rise in cytokine production upon LPS, confirming the role of intact CD44 and its activation in the cellular response to LPS. Several molecules could be responsible for CD44-ligation *in vivo*, including HA, osteopontin, and cytokines [8,40]. Engagement of CD44 activates the PI3K/AKT, MAPK/ERK, p38 MAPK pathways [29,41,42], which are induced upon LPS and mediate LPS-elicited NF-κB (PI3K/AKT, MAPK) and AP-1 (MAPK) activation, and CD14/TLR-4/MD-2-dependent LPS-uptake (p38 MAPK) [28,30-33,43,44]. Furthermore, CD44 is a fully competent phagocytic receptor that is able to trigger ingestion of large particles by macrophages. Vachon et al. showed that CD44-stimulation induces inside-out activation of CD11b/CD18 via Rap1 and suggest that ligation of CD44 by its ligands may serve as primary trigger for integrin activation, leading to enhanced phagocytosis [14]. Hence, we may hypothesize that CD44 activation augments NF-κB and AP-1 activation and LPS-uptake, thus increasing cytokine production.

CD44 can form a complex with TLR-4 [13]; this interaction is, however, not required for LPS-induced TLR-4 activation. In absence of CD44, LPS-treated BMM are still able of producing cytokines and activating the NF-κB pathway; in addition, TLR-4-inhibition completely blocks cytokine secretion in an equal way between the genotype-groups. We did, anyhow, observe that blocking p38 MAPK or PI3K/Akt, prior to exposure to LPS, diminishes cell response to LPS with a greater effect in WT BMM. Most importantly, when exposed to the inhibitors, LPS-stimulated WT BMM produced TNF-α at the same level as CD44 KO BMM treated with solely LPS. These results suggest that CD44, through activation of p38 MAPK and PI3K/Akt pathways, enhances TLR-4 signaling and, hence cytokines production (as discussed above). 

In the LPS-induced TLR-4 activation, serum proteins LBP and soluble (s) CD14 play an important role; CD44 can also be cleaved and found in plasma [8]. However, a possible role of soluble serum proteins, including sCD44, has been ruled out as CD44 KO cells stimulated with LPS in presence of homologous and heterologous WT serum did not shown significant differences in cytokine secretion (data not shown).

Current literature about the function of CD44 in host defense is quite controversial. The role of CD44 in inflammation appears dependent on the exposure as well as on the duration, intensity of, and timing after environmental challenge. Our data are in line with the reports of Hollingworth and of Hasan. The first study showed that macrophages and neutrophils of CD44 KO mice failed to recruit into lungs 24 hours after LPS-inhalation, and that CD44 KO macrophages displayed reduced ability to secrete TNF-α in response to LPS, reduced motility toward chemoattractants and reduced adhesion to vascular endothelia [16]. The report of Hasan demonstrated that CD44 contributes to pulmonary infiltration of neutrophils and lung damage in abdominal sepsis. Moreover, the authors showed that targeting CD44 prevented lung edema and tissue destruction [17]. Additionally, a genetic study of DNA samples of healthy or severely septic African Americans showed that the copy number variants of CD44 gene are associated with increased susceptibility to sepsis (Abstract 179.1_MeetingAbstracts.A2752, American Journal of Respiratory and Critical Care Medicine - ajrccm conference 2009).

Our results are not in agreement with the study of Kawana et al. entitled “CD44 suppresses TLR-mediated inflammation”. Here, using murine embryonic fibroblasts and BMM, the authors showed an increased pro-inflammatory cytokine production after 24 hours stimulation with 10ng/ml LPS in CD44 KO cells [45]. The dissimilarities in methodology compared to our *in vitro* assays consist in a different protocol to prepare BMM (5 days with M-SCF) and lower LPS concentration at one time-point. Even though we did not observe the same effect in 24 hours treated cells, the pro-inflammatory cytokine levels in plasma, kidneys, or BMM supernatants were similar between the genotype-groups at 24 hours. Another study showed that “endotoxin tolerance” can be induced in a CD44-dependent manner by injecting HA into mice prior to LPS administration [46]. We therefore speculate that at 24 hours WT cells undergo excessive stimulation by LPS, HA, and/or other ligands that subsequently triggers the immunoparalysis, which is typical of late stage sepsis [21]. Thus, WT cells might not further respond to stimuli and behave like CD44 KO cells. 

In conclusion, our study identifies CD44 as an auxiliary molecule in the initiation of the inflammatory response to LPS and highlights its contribution to inducing a kidney inflammatory state during endotoxic shock.

## Supporting Information

Figure S1 **Renal tubular damage, endothelial activation and expression of signaling molecules.** (**A**) Q-PCR analysis for expression of KIM-1 and (**B**) NGAL mRNAs in WT (white bars) and CD44 KO (black bars) kidneys; data normalized for TBP expression levels. (**C**) Quantification of tubular apoptosis rate; number of active caspase-3 positive tubular epithelial cells per HPF (x20). (**D**) Q-PCR analysis for expression of ICAM-1 normalized for TBP expression levels. (**E**) Digital analysis of renal expression of ICAM-1 at 2 hours, (**F**) phosphorylated (p)-AKT at 4 hours, and (**G**) phosphorylated (p)-p38 MAPK at 4 hours, detected by immunostaining on paraffin renal sections. Data shown as positive area percentage of total area. Mean + SEM, n=8, #=p<0.05 vs sham; in (**D**) all groups # vs sham.
(TIF)
