# Supplementary figures and images for: CD44-Deficiency Attenuates the Immunologic Responses to LPS and Delays the Onset of Endotoxic Shock-Induced Renal Inflammation and Dysfunction
Source: PLoS One. 2013 Dec 23;8(12):e84479. doi: 10.1371/journal.pone.0084479 (PMC3871539; doi:10.1371/journal.pone.0084479)

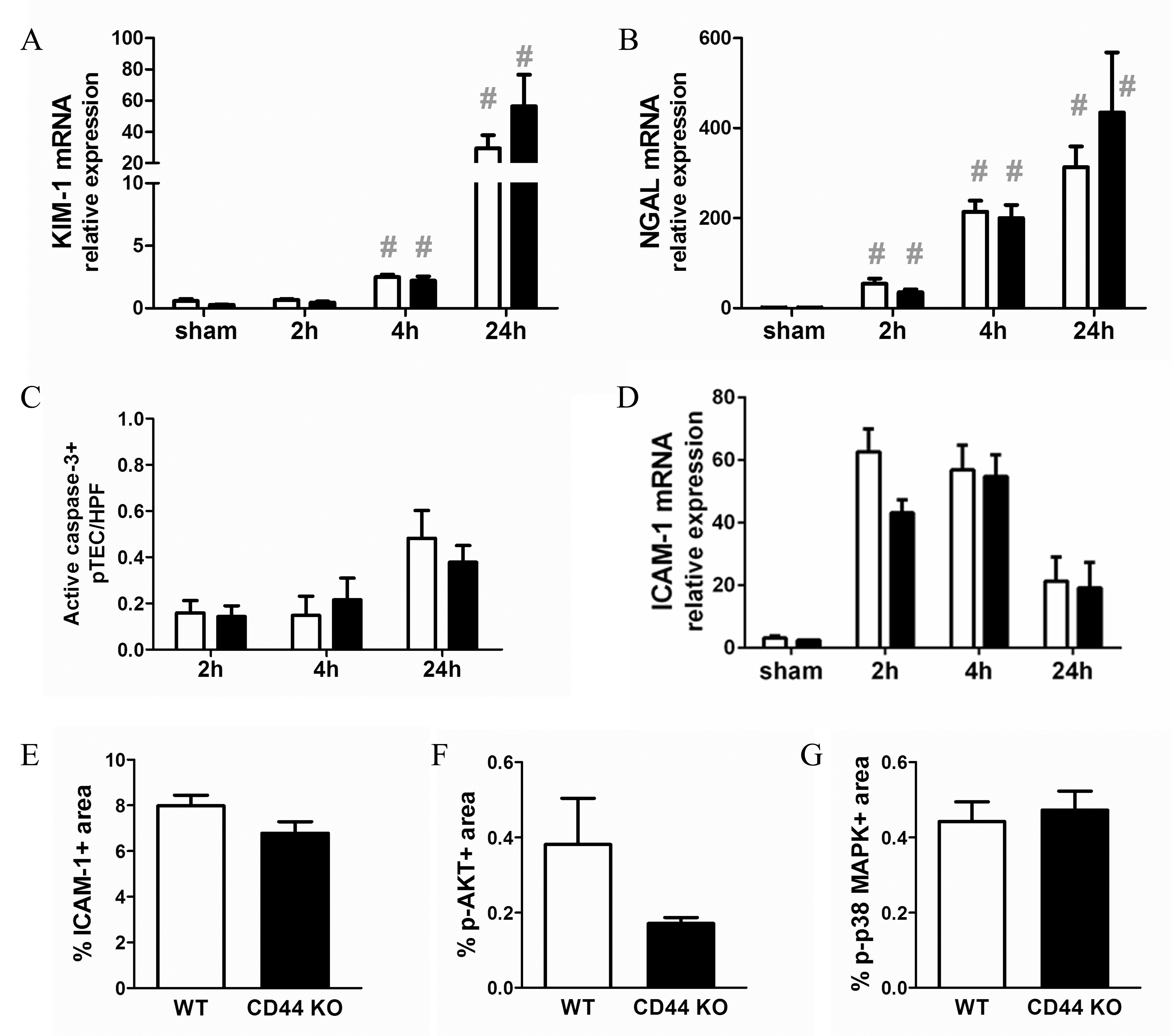

Supplement: Figure S1 — Renal tubular damage, endothelial activation and expression of signaling molecules. (A) Q-PCR analysis for expression of KIM-1 and (B) NGAL mRNAs in WT (white bars) and CD44 KO (black bars) kidneys; data normalized for TBP expression levels. (C) Quantification of tubular apoptosis rate; number of active caspase-3 positive tubular epithelial cells per HPF (x20). (D) Q-PCR analysis for expression of ICAM-1 normalized for TBP expression levels. (E) Digital analysis of renal expression of ICAM-1 at 2 hours, (F) phosphorylated (p)-AKT at 4 hours, and (G) phosphorylated (p)-p38 MAPK at 4 hours, detected by immunostaining on paraffin renal sections. Data shown as positive area percentage of total area. Mean + SEM, n=8, #=p<0.05 vs sham; in (D) all groups # vs sham. (TIF) [file pone.0084479.s001.tif]
